# Removal of Bullet from Right Ventricle

**Published:** 1916-04

**Authors:** 


### ABSTRACTS.

Have you ever felt that this department of the BUFFALO MEDICAL JOURNAL was lacking in abstracts of your specialty, or that such as were published lacked the expert judgement which you yourself could have exercised in selection and preparation? If not, you are more charitable than the editor has been to himself. If so, will you assist in abstracting from a few journals, and thus make this department more representative and more valuable? Incidentally, there are few forms of study more helpful to the worker than this.

**Removal of Bullet From Right Ventricle.** L. H. C. Birkbeck and H. M. W. Gray, Brit. M. J., Oct. 16, report a case of a soldier, X-ray showing bullet in or close to the lowest portion of the wall of the right ventricle. Operation under moderate dose of morphine, with local use of eucain, potassium sulphate and adrenalin, was successful but death occurred 4½ days later from multiple pulmonary infraction from clots in the ventricle.

